# Complications of Computer Tomography Assisted Radiofrequency Ablation in the Treatment of Osteoid Osteoma

**DOI:** 10.1155/2019/4376851

**Published:** 2019-05-15

**Authors:** Yunus Oc, Bekir Eray Kilinc, Sahin Cennet, Mehmet Metin Boyacioglu, Rodi Ertugrul, Ali Varol

**Affiliations:** ^1^Bagcilar Medilife Hospital, Istanbul, Turkey; ^2^Health Science University, Fatih Sultan Mehmet Training and Research Hospital, Orthopedics Department, Istanbul, Turkey; ^3^Sisli Hamidiye Etfal Training and Research Hospital, Radiology Department, Istanbul, Turkey; ^4^Sisli Hamidiye Etfal Training and Research Hospital, Orthopedics Department, Istanbul, Turkey; ^5^Kilis State Hospital, Turkey; ^6^Silopi State Hospital, Sirnak, Turkey

## Abstract

**Background:**

Osteoid osteoma (OO) is one of the most commonly occurring benign bone tumors. It constitutes 10-12% of benign bone tumors and 2-3% of primary bone tumors. In radiofrequency ablation (RFA) treatment, the cells of the tumor are thermally inactivated by the help of electrodes shaped like needles. In our study, we aimed to show the major and minor complications in patients undergoing RFA and to show what should be done to prevent these complications.

**Methods:**

The study was carried out as a prospective study on the follow-up of 87 osteoid osteoma patients treated between 2015 and 2017. The youngest of the patients was 1 year old and the oldest was 42 years old. The RFA procedure lasted 10 min on average, excluding anesthesia and preparation. All lesions were ablated at 90 degrees for 7 minutes with the heat increased gradually. All patients were followed up for 1 day in the orthopedics clinic.

**Results:**

Complications were observed in 7 patients. The lesions with the most complications were observed to be in the tibia, second-degree burns were seen in 2 patients, and superficial skin infection was observed in 2 patients. In 1 patient, the probe tip was broken and remained within the bone. Intramuscular hematoma was detected in 1 lesion located in the proximal femur. A complaint of numbness in the fingers developed in a lesion located in the metacarpus.

**Conclusion:**

Preventive measures should be taken before the procedure in order to prevent minor complications, and, for major complications, close follow-up should be done after the procedure and patients should be kept away from heavy physical activities for the first 3 months.

## 1. Introduction

Osteoid osteoma (OO) is the most common benign bone tumor. It constitutes 10-12% of benign bone tumors and 2-3% of primary bone tumors [1]. This tumor has a low growth potential and is usually less than 1 cm in diameter. Histologically, nidus was found in 85% of osteoid osteoma cases [2]. Osteoid osteoma can be observed in many bones in the body, but the principal locations are in the long bones of the lower extremities (femur, tibia, and fibula). It is common in children and young adults and the male to female ratio is approximately 4/1 [3].

In most cases, computed tomography (CT) is sufficient in determining the nidus and the sclerotic area around it. Magnetic resonance imaging (MRI) has not been shown to be superior to CT in the diagnosis of OO [4].

In the treatment of this tumor, classical surgical methods such as curettage, en bloc resection, and wide resection have been applied for many years and the success of classical surgery has been reported as 88-100%. However, new treatment methods were needed due to excessive complications such as avascular necrosis of the femoral head and fractures. Due to the high rate of complications (20-45%), long surgical duration, and frequent problems such as tissue damage, scar, and morbidity of classical surgery, there has been a rapid transition to minimally invasive treatment methods [5].

In radiofrequency ablation (RFA) treatment, the cells of the tumor are thermally inactivated by the help of electrodes shaped like needles. This electrode is placed in the center of the tumor with the help of CT images by opening a pathway in the bone. The location of the needle should be in a way to ensure the least possible complications and the best treatment. Since it is minimally invasive, the duration of hospital stay and the duration of rehabilitation after this treatment are significantly shorter than in classical surgery [6, 7].

In our study, we aimed to show the major and minor complications in patients undergoing RFA and to show what should be done to prevent these complications.

## 2. Materials and Methods

The study was carried out as a retrospective study on the follow-up of 87 osteoid osteoma patients treated between 2015 and 2017. The youngest of the patients was 1 year old and the oldest was 42 years old. The mean age was determined as 15.9 ± 8 years. The number of male patients was 57 and the number of female patients was 30. The procedure was completed by performing spinal anesthesia in 62 patients, general anesthesia in 15 patients, and peripheral block in 10 patients.  Table 1 shows the location of the lesions of the patients and the regions where synovitis and complications were observed. Synovitis occurring after the procedure, which osteoid osteoma was able to cause when it was located intra-articularly, was not considered as a complication, and it was evaluated as a separate symptom where the pain lasted longer.

The procedure lasted 10 min on average, excluding anesthesia and preparation. All lesions were burned at 90 degrees for 7 minutes with the heat increased gradually. Subcutaneous dextrose injection was applied to areas with subcutaneous fat tissue to prevent complications. Intermittent ice compression was performed to all patients immediately after the procedure. All patients were followed up for 1 day in the orthopedics clinic.

The mean follow-up period of the patients was calculated as 55,04 ± 22,36 weeks. Patients were called for controls at the 1st, 3rd, and 6th months. Patients followed up for at least 6 months were evaluated in the study. Patients with follow-ups fewer than 6 months were not included in the study.

## 3. Results

**Figure 1:**
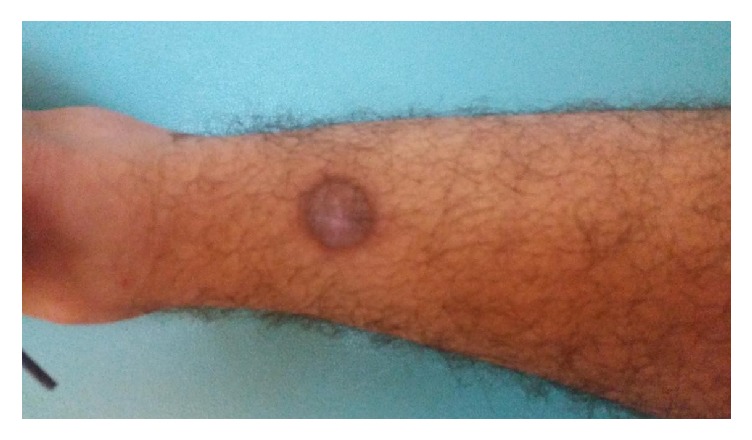
Second-degree burn in the lesion located in distal tibial anterior.

Complications were observed in 7 patients. The lesions with the most complications were observed to be in the tibia. Although subcutaneous dextrose injection was performed and ice was applied after the procedure in order to prevent complications in the tibia, second-degree burns were seen in 2 patients, and superficial skin infection was observed in 2 patients (Figure 1).

**Figure 2:**
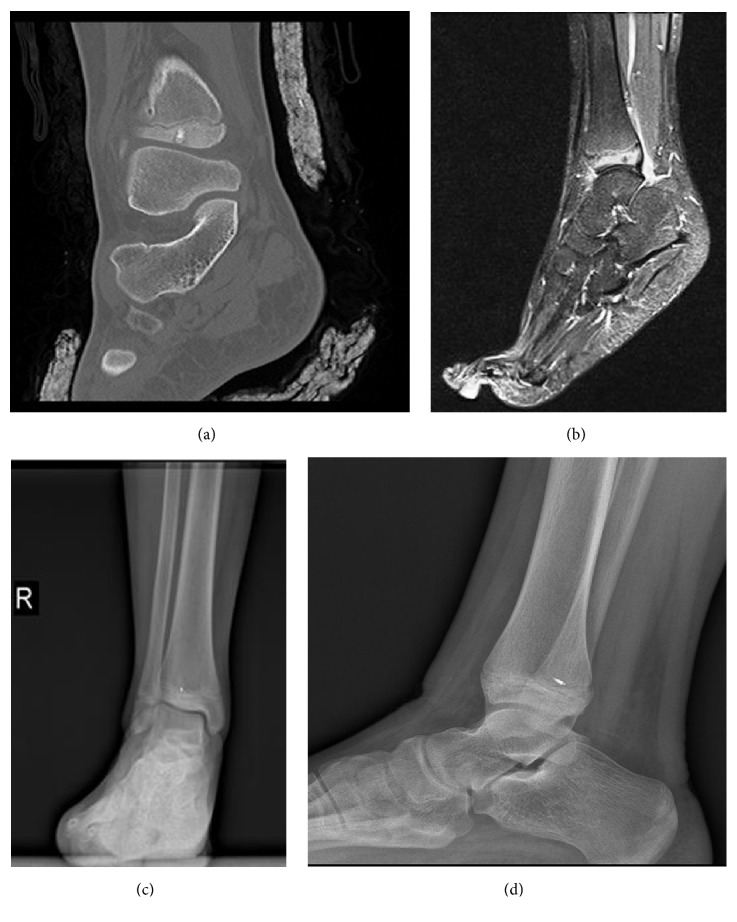
(a) and (b): CT and T2 MRI sections of the lesion located in distal tibial physis. (c) and (d): direct graph images of the probe tip broken after the procedure.

These patients were treated with oral antibiotherapy and topical ointments. In 1 patient, the probe tip was broken and remained within the bone (Figures 2(a), 2(b), 2(c), and 2(d)).

**Figure 3:**
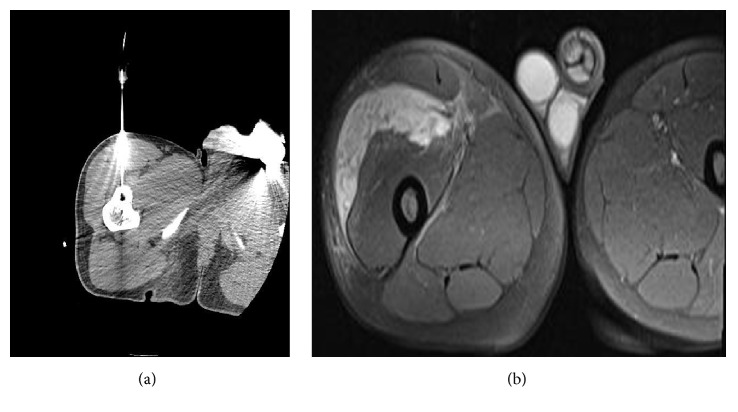
(a) CT image of the lesion located in the proximal femur during the procedure. (b) Hematoma on T2 MRI taken after the procedure.

The fractured part was not extracted because it would increase morbidity. Intramuscular hematoma was detected in 1 lesion located in the proximal femur. It was not related to an arterial injury. Hematoma regressed smoothly after 6 weeks of follow-up and treatment (Figure 3). A complaint of numbness in the fingers developed in a lesion located in the metacarpus. After 6 weeks of follow-up, the complaint was regressed.

**Figure 4:**
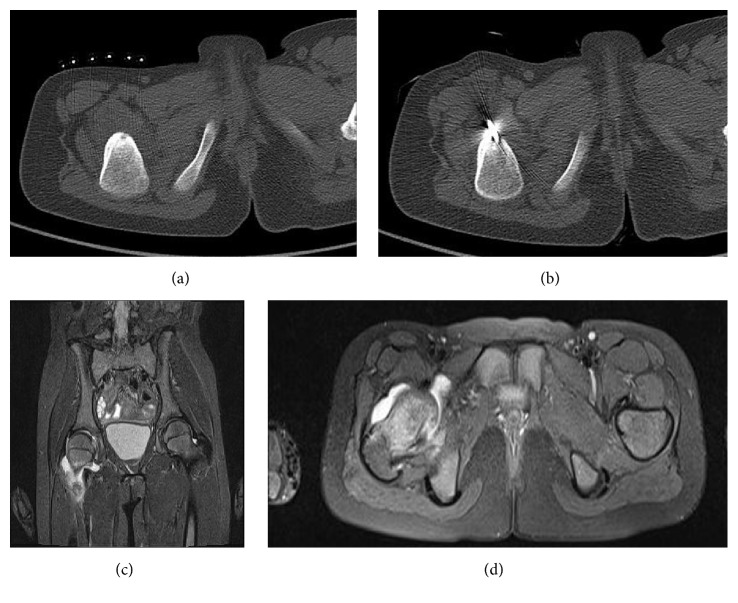
(a)-(b) 8-year old girl patient with CT image of the lesion in right femoral neck. (c)-(d) T2 MR images at 4th week after the procedure.

The intra-articular OO itself may occur as a synovitis complication; therefore the synovitis was not considered as a complication in the follow-up period. Apart from these complications, it was seen that pain might continue in the intra-articularly located osteoid osteoma lesions due to reactive synovitis in the joints after the procedure, which was different from the pain before the procedure. It was observed during follow-ups that this pain could last up to 3 months (Figure 4).

## 4. Discussion

Radiofrequency ablation has been proven to be a safe and effective treatment for the treatment of OO with numerous studies in recent years [8, 9]. The fact that RFA treatment has a lower complication rate and less morbidity and requires a shorter hospitalization time compared to percutaneous drilling and surgical excision made it the first choice [10, 11].

The greatest advantage of RFA treatment is that it is minimally invasive compared to percutaneous drilling and surgical resection as it deteriorates bone integrity less. With the increased application of RFA as the primary treatment of choice due to its advantages over the other treatments, the number of procedures has increased rapidly over time [12].

Patients with OO started to be treated by two different branches (orthopedics and radiology) and differences in patient follow-up and treatment could be encountered between these two branches. This can cause leaving out complications, especially after RFA treatment. As a matter of fact, only 5 (0.9 %) complications were reported in a paper from the radiology clinic where 557 patients were treated, and 7 (6.1%) complications were reported in a paper from the orthopedics clinic where 87 patients were treated, which clearly reveals the difference of the working styles of two different clinics [12, 13].

Skin burns appearance is the most common complication in RFA treatment. In the case of Finstein et al., they reported skin burns with necrosis 1 cm in diameter after thermocoagulation applied to tibial osteoid osteoma [14]. In a study by Bourgault et al., even though cold saline solution was poured on the skin during heat application, late recovery was reported in burns and wounds in 3 cases [13]. In the series of 86 cases by Nijland et al., skin burns were detected in 2 patients [15]. In the 18-case study by Karagoz et al., skin burns were detected in 1 patient [16]. We detected second-degree skin burns in 2 patients. There is very little soft tissue between the bone and the skin particularly in tibial lesions, which poses a great risk in terms of burns. Although subcutaneous dextrose was applied in tibial lesions to prevent burns, skin burns were observed in 2 patients. These patients were treated with local burn creams and prophylactic oral antibiotics (Figure 1).

More severe burns have been reported due to the effect of heat. In the series of 21 cases published by Earhart et al., they reported skin burns as well as vastus lateralis muscle burns in their 7-year-old patients. Preventive measures and temperature adjustment are especially important in pediatric patients in order to protect against major complications requiring surgical operation [17].

Different applications can be made to prevent tissue burns. Martel et al. performed ablation with a cold tip probe to their osteoid osteoma patients to avoid burn complications [18]. Another method to protect tissues from burn complications is to protect the tissues with carbon dioxide, which is applied in ablation of liver and kidney tumors [19].

When RFA is performed, it is sometimes necessary to cross an intense sclerotic bone tissue at the stage of reaching the lesion and then place the probe in the lesion site. During this process, cannula and probe fractures are seen and sometimes these intraosseous fractures can remain in the bone. In a series of 77 cases by Rehnitz et al., one patient had a cannula fracture requiring secondary hospitalization and it was extracted with a small operation [20]. Since the procedure is in the sclerotic bone tissue, fractures of the instruments used for entry or the probe are frequently encountered [6, 12, 21].

In one of our patients, the probe tip remained within the bone after developing a probe fracture. As it was a tibial distal end physis lesion, no extraction was performed considering physis damage. It was observed in the follow-up that the patient had a different pain after the pain had completely disappeared. The patient was followed up and treated for pain due to foreign body reaction, and no recurrent procedure was performed. Pain completely regressed in the 6th month (Figure 2).

Close follow-up is required for patients whose pain completely disappears on postoperative 2nd day but who have pain of different characters afterwards. MRIs taken early in these patients are very valuable in terms of showing both bone and soft tissue lesions. An intramuscular hematoma was detected in our patient who underwent ablation for a proximal femoral lesion. The patient, who was followed up and treated, became completely painless at the 1st month (Figure 3). If hematoma in the tissue is not detected and is untreated, it may form into an abscess and become a major complication requiring surgery. Gebauer et al. reported a soft tissue abscess that required surgery after 3 months of follow-up in their 59-case series [22].

After RFA, a considerable amount of fracture complications occurs and usually requires surgical treatment, although we did not encounter one in our own series. Another major complication in Earhart's series was subtrochanteric fracture that developed at 9 weeks after the procedure. The fracture was surgically fixed with plaque and treated [17]. Gebauer et al. reported tibial shaft fracture in their series 2 months after RFA for tibial OO. The fracture was surgically treated with intramedullary nails [22]. Nijland reported a fibula fracture that occurred during the procedure in his series and stated that it recovered without the need of surgery [15]. Bonicoli et al. concluded that the subtrochanteric fracture forming 7 years after RFA was due to the changes occurring in the bone after ablation as it was in the area of operation and as it formed due to simple trauma. We particularly warned our patients with lower extremity lesions to avoid difficult exercises and contact sports for 3 months after the procedure to prevent formation of fractures.

After RFA, pain may be present in the joints in osteochondral lesions due to reactive synovitis and some patients may be referred to a repeat procedure in the early period. This maybe occurred by the way of the cannula with soft tissue connect. Patients who are relieved of pain in the first week but have a different style of lighter pain should be examined in terms of reactive arthritis.

We could not find clear information about the frequency of postablation reactive arthritis in the literature. In our series, we observed development of reactive arthritis in 4 patients (4.5%). In all of these patients, pain completely regressed in the first 2 days, and 7-10 days later, pain started in the joint treated more lightly (Figure 4). After increased intra-articular fluid was observed in the MRIs of the patients, laboratory tests (CRP, WBC, and sedimentation) were requested from them for differential diagnosis of septic arthritis. The patients, for whom septic arthritis was ruled out after the examinations, were followed up with anti-inflammatory treatment. The pain of all of the patients completely ceased in the first 3 months.

## 5. Conclusion

Among the treatment methods for osteoid osteoma, the complications of RFA are less than percutaneous drilling and open surgery, but the complications of RFA are not considerably few even though it is minimally invasive. Cryoablation is another ablation method in the treatment of osteoid osteoma. Complications of cryoablation are not well known such as RFA because the complication rates of cryoablation are low and it is used less. Complications of it such as fractures, abscesses, and deep burns are complications that require surgery and seriously increase morbidity. Preventive measures should be taken before the procedure in order to prevent minor complications, and for major complications, close follow-up should be done after the procedure and patients should be kept away from heavy physical activities especially to prevent fracture complications for the first 3 months.

Patients whose pain regresses and in whom then a lighter one starts in the joint treated should not be directed to repeat operation directly considering recurrence in a short time. Such patients should be evaluated with MR and laboratory tests for reactive arthritis and should be followed up for at least 3 months.

## Figures and Tables

**Table 1:** Location of the lesions.

Region	Number	Recurrence	Synovitis	Complication
Femur	44 (50,5%)	--	2	1
Tibia	24 (27,5%)	--	1	5
Pelvis	6 (6,9%)	--	--	
Fibula	3 (3,4%)	--	--	
Humerus	2 (2.3%)	1	--	
Talus	2 (2.3%)	--	1	
Radius	1 (1.14%)	--	--	
Scapula	2 (2.3%)	--	--	
Hand	1 (1.14%)	--	--	1
Foot	2 (2.3%)	--	--	

## Data Availability

All data created during this research is openly available from the corresponding author upon request.
